# An open-source tool for analysis and automatic identification of dendritic spines using machine learning

**DOI:** 10.1371/journal.pone.0199589

**Published:** 2018-07-05

**Authors:** Michael S. Smirnov, Tavita R. Garrett, Ryohei Yasuda

**Affiliations:** 1 Neuronal Signal Transduction, Max Planck Florida Institute for Neuroscience, Jupiter, Florida, United States of America; 2 Neuroscience, Oregon Health and Science University School of Medicine, Portland, Oregon, United States of America; Science and Technology Facilities Council, UNITED KINGDOM

## Abstract

Synaptic plasticity, the cellular basis for learning and memory, is mediated by a complex biochemical network of signaling proteins. These proteins are compartmentalized in dendritic spines, the tiny, bulbous, post-synaptic structures found on neuronal dendrites. The ability to screen a high number of molecular targets for their effect on dendritic spine structural plasticity will require a high-throughput imaging system capable of stimulating and monitoring hundreds of dendritic spines in various conditions. For this purpose, we present a program capable of automatically identifying dendritic spines in live, fluorescent tissue. Our software relies on a machine learning approach to minimize any need for parameter tuning from the user. Custom thresholding and binarization functions serve to “clean” fluorescent images, and a neural network is trained using features based on the relative shape of the spine perimeter and its corresponding dendritic backbone. Our algorithm is rapid, flexible, has over 90% accuracy in spine detection, and bundled with our user-friendly, open-source, MATLAB-based software package for spine analysis.

## Introduction

Structural changes in dendritic spines, tiny postsynaptic protrusions on the dendritic surface of neurons, are considered to be the basis of synaptic plasticity [1] and are known to be important for learning and memory [2]. Dysfunctions in synaptic plasticity are a feature of affective disorders, neurodegenerative diseases, and aging-associated cognitive decline [1].

Recent advances in photostimulation and imaging techniques have made it possible to visualize the morphological and molecular changes in individual spines with high time resolution. Two-photon laser-scanning microscopy in live brain tissue is often used due to its relatively low scattering and precise localization in deep samples [3]. Furthermore, two-photon microscopy can be combined with glutamate uncaging, resulting in targeted photoactivation and plasticity in individual dendritic spines [4]. However, the process of finding, imaging, and analyzing changes in individual dendritic spines is cumbersome and time-consuming. Therefore, the identification of dendritic spines needs to be automated.

Recently, several approaches to semi-automated identification and analysis of dendritic spines have been described [5–10]. These methods have the potential to greatly reduce the amount of effort required for large-scale spine counting and analysis, but are often optimized to a specific cell type, imaging technique, or magnification. Since the majority of spine segmentation algorithms are designed to be used for post-hoc analysis rather than to assist with live imaging, they may require large amounts of computing time and always rely on human input for error correction. Furthermore, variations in image intensity, background signal, and spine length must be accounted for by manual optimization of program settings. Therefore, the application of these algorithms to assist with live spine imaging under varying physiological conditions proves prohibitively difficult.

To reduce errors due to sample variability, some spine identification techniques incorporate machine learning techniques [5, 11]. Since differences in microscopes, fluorescent markers, and spine morphologies lead to variability in how spines are visualized, complex machine learning algorithms such as neural networks and deep learning often require enormous amounts of labeled training images (>10,000), while simpler classifier techniques lack the ability to properly capture the amount of features required to identify spines.

Here we provide a user-friendly tool to analyze, label, segment, and automatically identify dendritic spines. We use a machine learning approach to dendritic spine identification which is highly adaptable to any fluorescent imaging setup. By using adaptive thresholding, we identify neuronal dendrites regardless of background noise and signal intensity. Next, we train a neural network to identify spines based on the position of perimeter pixels relative to the dendrite and spine backbone, as well as the fluorescence intensity along the spine backbone. Our approach is fast and works with a training data set of as few as two thousand images which can be labeled within a few hours using our semi-automated labeling software. Furthermore, our software can be easily adapted to unique imaging setups, and is freely available in open-source MATLAB code.

## Image acquisition

### Tissue preparation

To create an algorithm able to detect dendritic spines within a variety of morphologies, the images used for analysis were collected from a variety of genotypes. Organotypic hippocampal slice cultures were prepared as described previously [12] from p4-p6 mice were cultured for 10–12 days before transfection. A biolostic particle delivery system (Helios® Gene Gun System, Bio-Rad) was used to introduce fluorescent GFP labels to obtain sparse transfection of neurons. Two to six days after transfection, neurons in sparsely GFP-labeled CA1 hippocampal regions were chosen for imaging. Individual spines in the striatum radiatum on secondary apical dendrites were chosen for observation.

### Animals

Wild-type C57BL/6J were purchased from Charles river laboratories, and conditional knockout (cKO) lines were generated for IGF1 Receptor and Insulin Receptors as using standard knockout techniques. Animals were housed on a 12 hour light cycle with a room temperature of 74°F, 50% humidity, with Harlan 7092 ¼” corn cob bedding. P4-p6 pups were taken from mothers housed individually in Tecniplast® ventilated cages. Pups were sacrificed using decapitation. This study, including all animal procedures, was approved by the Max Planck Florida Institute for Neuroscience Animal Care and Use Committee, in accordance with guidelines by the US National Institutes of Health. Max Planck Florida Institute has been AAALAC Accredited since June, 2014.

### Microscopy

Imaging was done on a custom built, two-photon microscope controlled by Scanimage and modified to allow for automated, multiposition image collection [13, 14]. Dendritic spines were imaged over ~1 hour using a 60X objective and 30X or 15X galvanometer-scan zoom (image field ~8x8 μm or 16x16 μm). One 5 μm Z-stack was collected over five Z-planes at each imaging position per minute. Each image was acquired at 128x128 pixels, resulting in a resolution of ~ 15 pixels per μm in both X and Y.

### Image analysis

**Fig 1:**
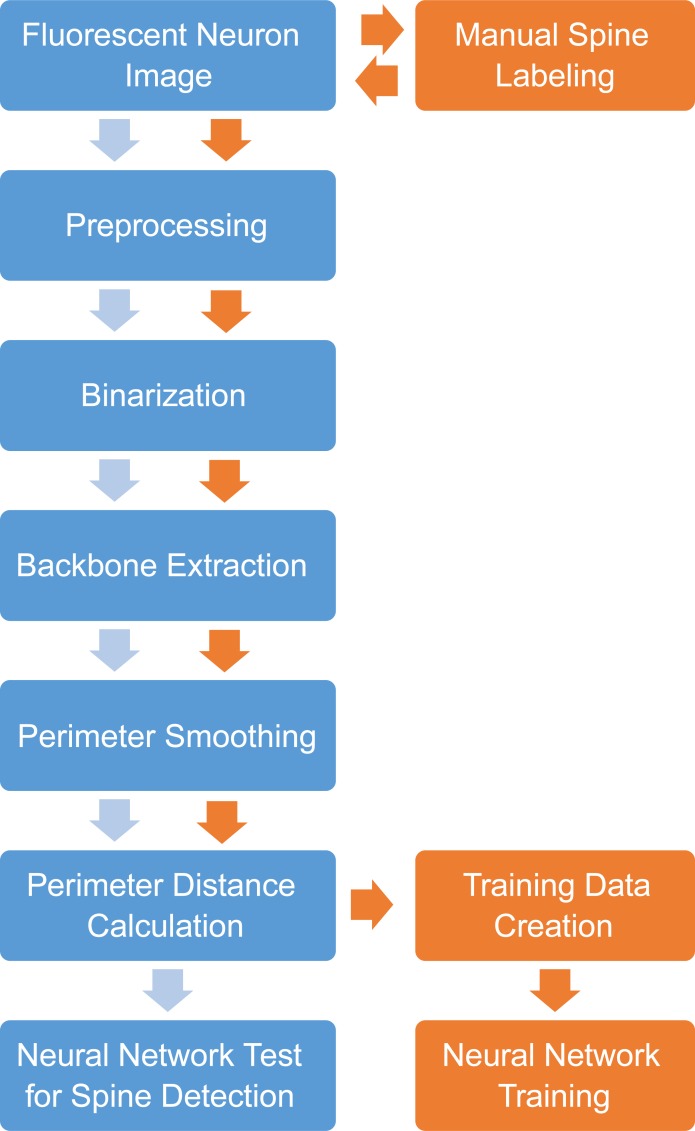
Image processing workflow for automated identification of dendritic spines. Orange: Spine locations are labeled in each image prior to automated segmentation. Extracted feature vectors are used to train a neural network using a scaled conjugate gradient propagation algorithm. Blue: Novel images are preprocessed, segmented, and feature vectors are extracted. Feature vectors are used to evaluate identify potential dendritic spines using the previously trained neural network.

The image processing workflow for feature extraction is illustrated in Fig 1. First, spine locations are labeled in each image, and images are automatically segmented. After segmentation, individual feature vectors consisting of 221 values were used to train a neural network using a scaled conjugate gradient propagation algorithm [15]. Once trained, the neural network was used to evaluate whether feature vectors from newly segmented images represent spine or non-spine locations. All code was written in MATLAB and is freely available at https://github.com/mikeusru/Braintown. All functions have been tested in MATLAB 2016b and require the Image Processing, Neural Network, and Statistics and Machine Learning toolboxes. All training data has also been made available [16].

### Preprocessing and binarization

**Fig 2:**
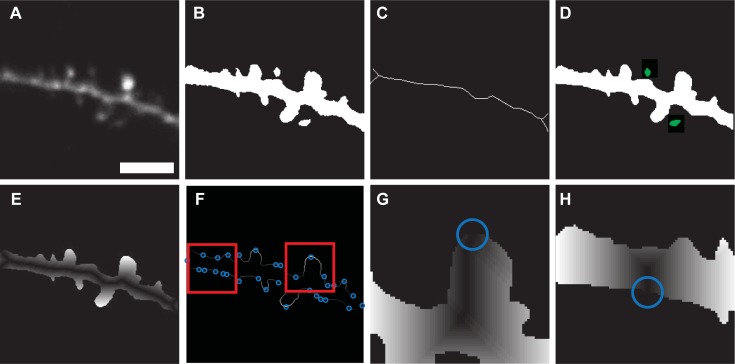
Steps in image segmentation. A. Original Image. B. Thresholding using Otsu’s global method, followed by adaptive thresholding and binarization. C. Backbone Extraction D. Identification of removed spines. E. Geodesic distance transform using dendrite backbone as seed location F. Identification of potential spine locations by local maxima along perimeter. G, H. Local geodesic distance transforms for each individual potential spine point, using spine backbone (shortest path between local maxima and dendrite backbone) as seed point. Scale bar = 5 **μ**m.

Once an image is loaded (Fig 2A), pixels are converted to grayscale floating-point numbers ranging between 0 to 1. Noise is removed using a standard 2D median filter. Highest-probability background is identified using Otsu’s method of globally thresholding [17]. To ensure no relevant pixels are lost, the global threshold value is reduced by 70%. The average pixel below the background threshold is then subtracted from the image. Next, an adaptive image threshold is computed using local first-order statistics with a neighborhood size of 10x10 μm. Any resulting holes smaller than 0.5 μm^2^ are filled. Ideally, the resulting binary image (Fig 2B) includes only regions of neuronal tissue.

### Backbone extraction

The backbone of the dendrite (Fig 2C) was identified by thinning the binary image until all structures had a thickness of no more than one pixel, and then removing any branches which didn’t belong to the dendrite. After skeletonization [18], the presence of dendritic spines and noise within the image causes a significant number of spurious branches and loops which are not representative of the dendrite itself. Loop artifacts were removed by filling in all areas smaller than 0.5 μm^2^ and undergoing a second round of skeletonization. Any isolated segments where p<M were removed, where p was the amount of pixels in the segment, and M was the minimum branch length (2 μm) multiplied by the number of pixels per μm. The remainder of the spurious segments were removed by a recursive trimming algorithm adapted from Cheng et al. [7]. Basically, endpoint pixels were iteratively removed from the skeleton and added to a set of deleting templates through the use of a nested loop. If the iteration did not add to the deleting template, then the deleting template was permanently removed from the skeleton. The code structure is presented below:

1. Initialize m = 1
2. Repeat until m = M
  - Initialize removed segments = blank
  - Repeat m times:
    - Find skeleton endpoints, ignoring those near border
    - Add skeleton endpoints to removed segments
  - Remove skeleton endpoints from skeleton
  - Restore any removed segments that have m pixels
  - m = m + 1

After trimming, the backbone often retained some small kinks leftover from the initial skeletonization process. As these kinks could introduce artifacts in the later perimeter distance calculation, they were removed by a custom smoothing algorithm also adapted from Cheng at al. [7]: First, all branch points belonging to the initial, untrimmed skeleton were located along the dendrite backbone. Next, the branchpoints were dilated by M/4 to include all local backbone pieces which might belong to a kink. Finally, these kinks were removed, and the resulting line endpoints connected, resulting in a smooth backbone segment.

### Surface smoothing

**Fig 3:**
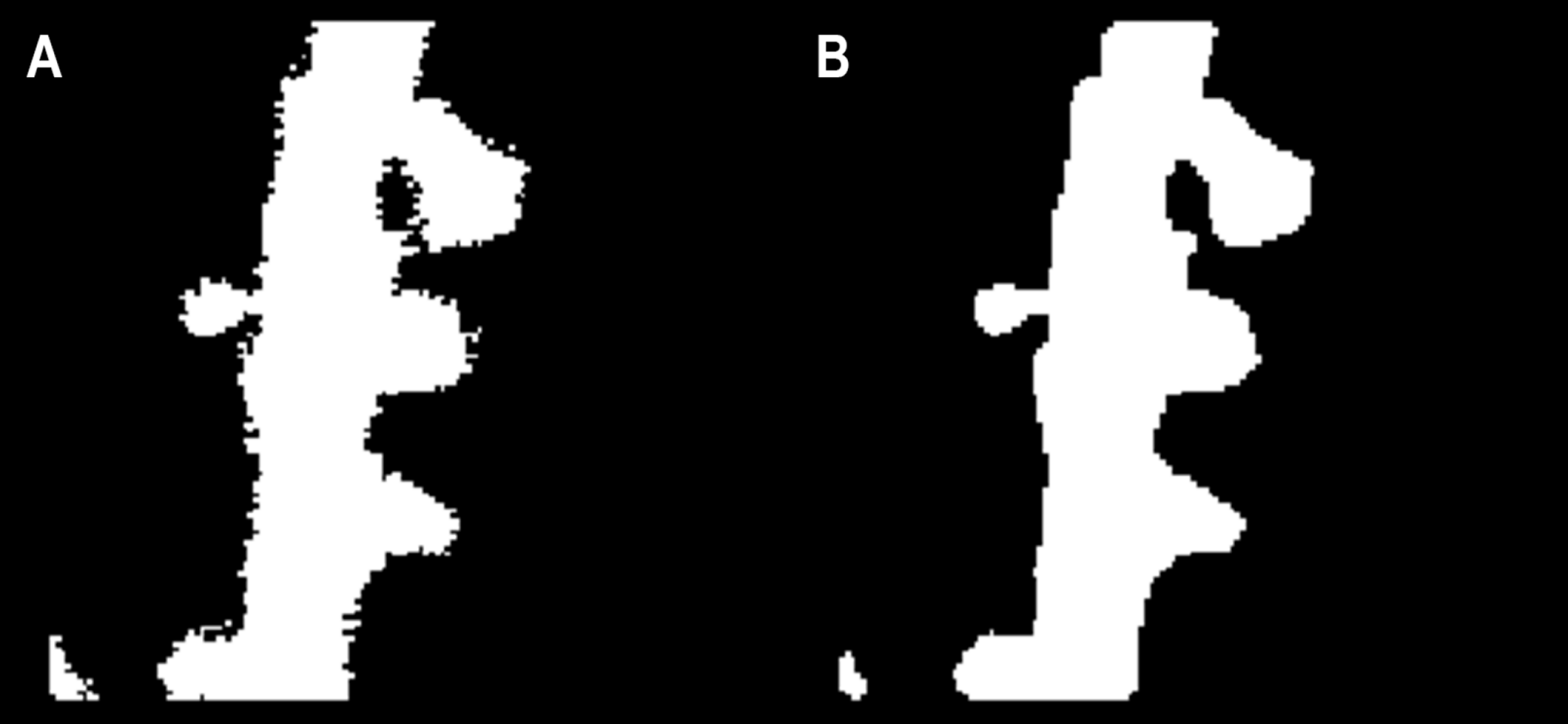
Surface smoothing of binary image. (A) Initial binary image contains a significant noise around its perimeter. (B) Surface-smoothed image lacks kinks, as well as spurious or diagonally–connected pixels.

To isolate individual segments of the cell perimeter to be used as features for spine detection, the surface of the binary object needed to be smooth, lacking any spurious pixels or diagonally connected regions. Smoothing was achieved using an array of morphological operations on the binary image. First, a majority operation [19] set a pixel to 1 if five or more pixels in its 3x3 neighborhood are 1s, otherwise the pixel is set to 0. Next, the image is morphologically opened, closed, and opened again using a 3x3 structuring element of ones. Pixels connected to fewer than three other pixels were removed, and a diagonal fill was used to eliminate any 8-connectivity of the background, essentially transforming diagonal connections into right angles. The binary objects were then thickened by adding a one-pixel width border, as long as that border did not form a new connection with a neighboring border. An example of the result attained through surface smoothing can be seen in Fig 3.

### Identification of disconnected spines

As the purpose of our algorithm was to find spines which were obviously attached to the dendrite, small objects which became disconnected from the dendrite (Fig 2D) during the surface smoothing step were categorized using k-means clustering, but were ignored from the neural network training data. In any individual image, the signal to noise ratio was calculated in objects that were within distance M from the dendrite backbone. Each respective signal was collected from the pixels in the original image (Fig 2A) which overlapped with the object, while noise was calculated using pixels in the bounding box of the object minus the pixels within the object. If more than two objects were detected, spines were identified using k-means clustering of the signal to noise ratios into two clusters. The group with a larger average signal to noise ratio were identifies as spines, while others were non-spine objects. All objects detached from the main dendrite structure were ignored for the remaining calculations.

### Perimeter feature extraction

**Fig 4:**
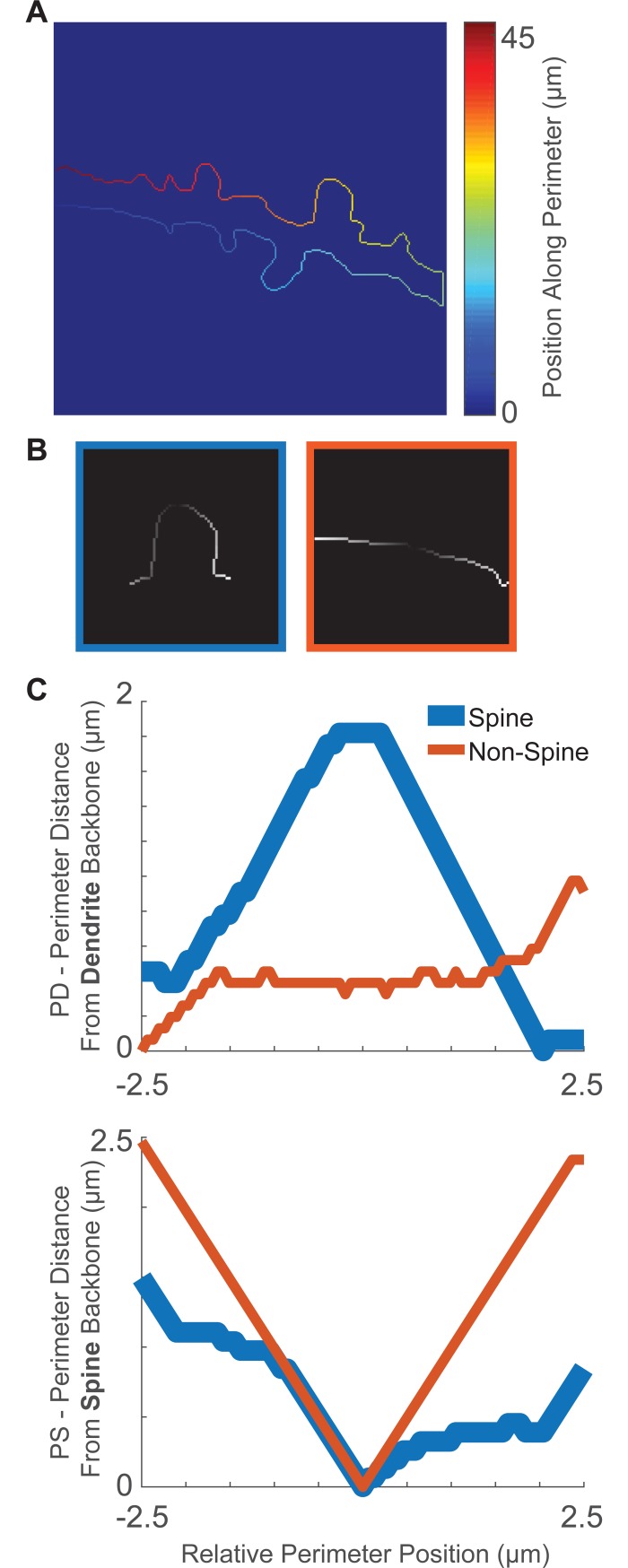
PD and PS feature vectors. (A) Relative position values, in **μ**m, assigned to each pixel starting from a random seed and following a single direction. (B) 5 **μ**m perimeter segments are extracted at each potential spine point to create PS feature vectors. Brighter pixels indicate higher distance from spine backbone. Left box (blue) is a true spine, right box (orange) is a non-spine. (C) Perimeter feature vectors represent respective pixel values (y axis) and pixel position (x axis).

Three feature vectors were used for neural network training and spine identification: perimeter distance from dendrite backbone (PD), perimeter distance from spine backbone (PS), and fluorescence intensity along the spine backbone (IS). The location of each feature vector, as well as the individual values of PD features, were quantified based on a geodesic distance transform [20] of the binary image of the dendrite, using the dendrite backbone as a seed location. Thus, the value assigned to each connected pixel represents its relative distance from the dendrite backbone (Fig 2E). The central position of each feature vector was assigned by finding local maxima along the perimeter of the geodesic transform (Fig 2F), and a geodesic distance transform of the perimeter itself (Fig 4A) served to organize all perimeter pixels into relative locations. Vectors were all standardized to contain a minimum value of 0 to reflect the shape of the spine while ignoring dendrite thickness. Each PD feature vector represents a 5 μm segment of pixel values along the edge of the geodesic transform (Fig 4C).

Unlike PD features, which represent distance from the dendrite backbone, PS features represent distance from the spine backbone. The spine backbone was identified as the shortest path between the feature origin point on the perimeter (Fig 2F, 2G and 2H) and its nearest point on the dendrite backbone with the help of a fast marching algorithm [21]. A geodesic distance transforms was calculated using the spine backbone as a seed (Fig 2G and 2H), and PS features are represented as a 5 μm segment of pixel values along the resulting perimeter (Fig 4B and 4C).

PD and PS features were arranged based on their respective position along the perimeter. To minimize the amount of necessary training data, position information was defined by a single value as the directional distance along the perimeter from the center of the feature origin. To assign position values, a closed-loop perimeter was first cut at a random point. A geodesic distance transform, with one endpoint as a seed, was then used to assign a single value to each pixel (Fig 4A). As a result of the transform, each consecutive pixel was assigned a value based on its travel distance from the seed pixel. Prior surface smoothing (Fig 3) was exceptionally important for this distance transform to work properly, since any kinks or loops in the perimeter would result in duplicate position values. By assigning these position values to each perimeter feature, we translated 2D perimeter images (Fig 4B) into 1D arrays of feature-specific values (Fig 4C). Finally, since the amount of pixels in a 5 μm segment varied based on the resolution of the initial image, PS and PD feature vectors were standardized by interpolating to 100 values each.

**Fig 5:**
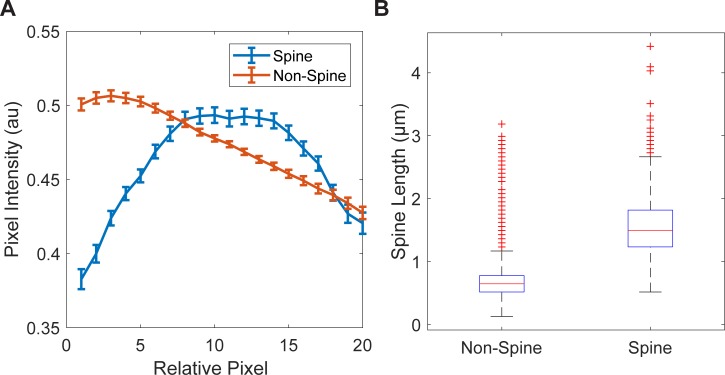
Length and Intensity of the spine backbone. **(**A) Average pixel intensity in pixels taken along line connecting tip of potential spine to closest point in dendrite backbone. (B) Average length of spine backbone in pre-labeled Spine and Non-Spine objects.

Features in the IS group were assigned using pixel positions from the spine backbone, and pixel values from the original image. The resulting feature vector represents a line of intensity values starting at the dendrite backbone and finishing at the tip of the spine. Due to the spine backbone varying in length, each group of values was interpolated to 20 features (Fig 5A), while the 21^th^ feature represented the original spine backbone length in μm (Fig 5B).

## Network training

PD, PS, and IS feature sets consisting of a combined 221 values were used to train a neural network using a scaled conjugate gradient propagation algorithm [15] from the MATLAB (2016b) Neural Network Toolbox using default parameters. Weights and biases were initialized randomly between -1 and 1. To elicit the highest accuracy in spine categorization while keeping training and classification times manageable, the network was configured to have a single hidden layer with 20 nodes. Features sets were classified as either spine or non-spine. To label training data, we designed an application which allows users to rapidly identify dendritic spines by clicking on their location in an image. A 1x1 μm box was then drawn around each identified spine. Boxes that were within 1.5 μm from the image border were ignored to avoid edge artifacts. Boxes which overlapped with a disconnected blob (Figs 2 and 1D) were ignored as well. Feature sets were classified as spines if their point of origin was inside the box.

## Software design

**Fig 6:**
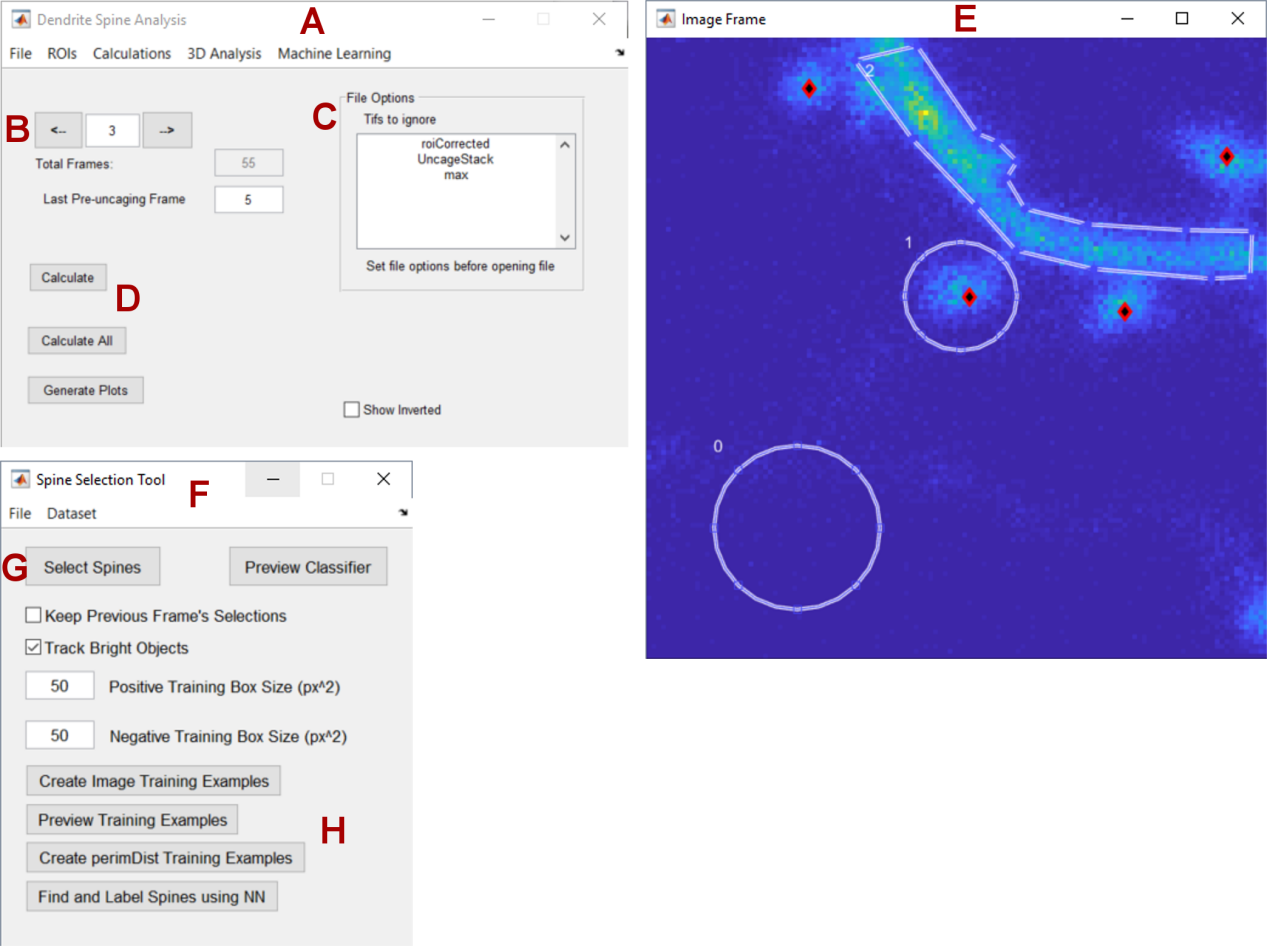
Graphical user interface to label and measure image regions. (A) Base dendrite analysis window responsible of loading/saving data, (B) switching between images, (C) avoid particular files, and (D) running calculations on ROI area over time. (D) Image preview window for drawing polygonal/circle ROIs, and identifying individual spines (red/black rhombi). (F) Spine selection and machine learning tool allows toggling of (G) spine selection mode, semi-automated spine tracking, (H) gathering and previewing of training data, and spine finding using a trained neural network.

To make our tools accessible to users who may lack any significant coding expertise, we built a straightforward front-end user interface for viewing, analyzing, labeling, and segmenting images of dendritic spines in MATLAB (Fig 6). The main window (Fig 6A) allows users to load either individual images or image sets, browse through the loaded data (Fig 6B), and restrict certain files from being loaded (Fig 6C). Drop-down menus also let users perform common calculations such as 3D projection on Z stacks and drift correction on timelapse image sets. Users can draw circular or polygonal ROIs on the image (Fig 6E) to calculate changes in spine volume over time (Fig 6D). While remaining compatible with variable data sources, this program is particularly tuned to analyze data collected using our automated multiposition imaging system [14]. A semi-automated spine selection tool for labeling training data is also provided (Fig 6F). Users can enter spine selection mode (Fig 6G), where clicking on the image frame will label and store the local coordinates of each spine (Fig 6E). Users have the option to track spines through brightness, where given a timelapse image set, spine coordinates will automatically update to their new closest position. Finally, the spine selection tool allows users to train, preview, and test a neural network for its capability to find dendritic spines (Fig 6H).

**Fig 7:**
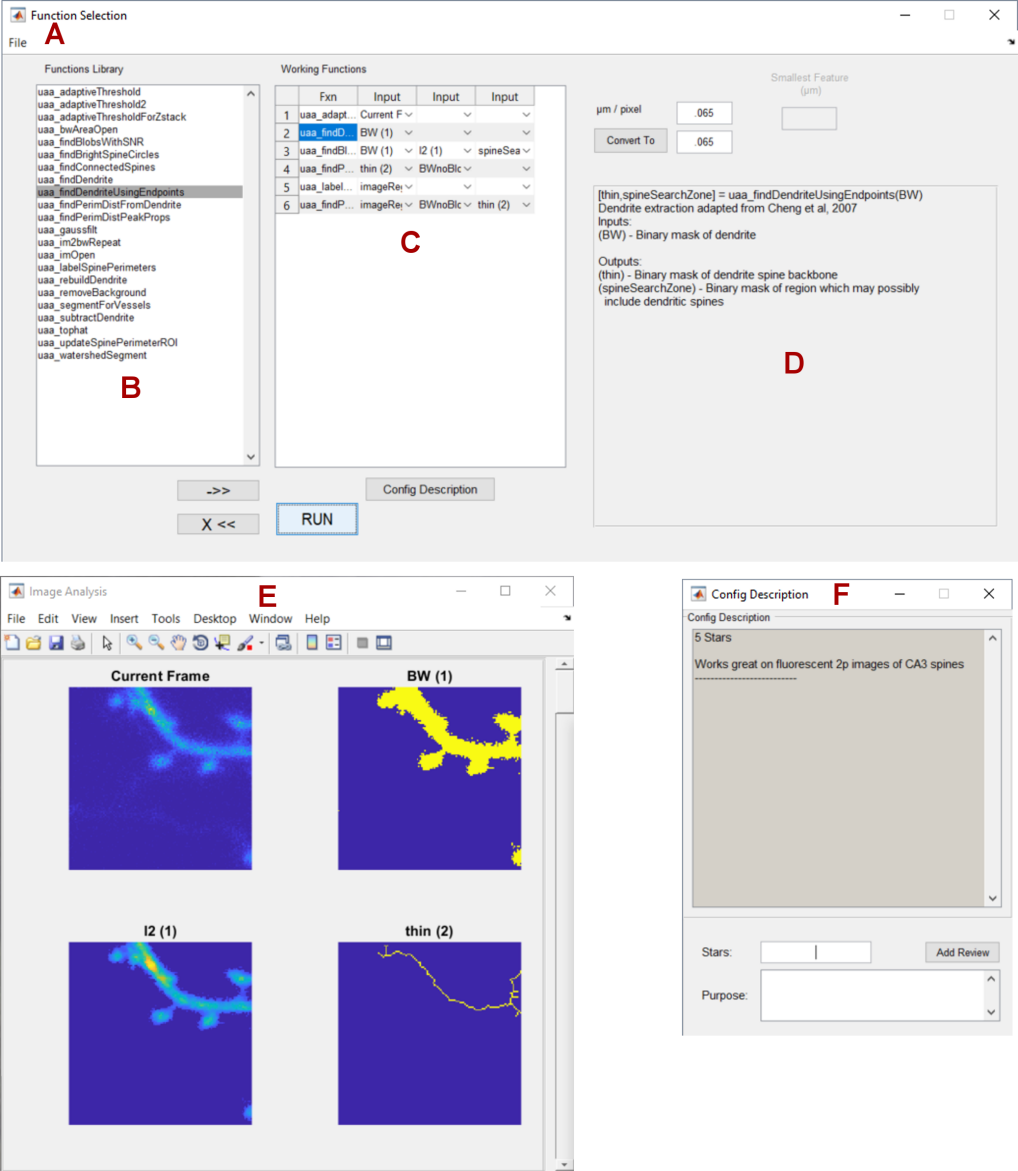
Graphical user interface for image segmentation. (A) The function selection window handles the available functions and their configuration. (B) All available functions are displayed in the plugin repository. (C) Selected functions and their inputs are selected in the current configuration space. (D) A short tutorial for each individual plugin is displayed upon selection. (E) Outputs of each plugin are previewed in a separate, scrollable window. (F) Plugin configurations can be saved, shared, and rated between multiple users.

To create a powerful yet user-friendly system for image segmentation, we created a modular interface where users can manually select, customize, evaluate, and share plugins and configurations without any coding experience (Fig 7). A function selection window (Fig 7A) loads all of the plugins from a local plugins folder and displays them in an alphabetized list (Fig 7B). Each plugin serves as a step in the image segmentation, analysis, or feature extraction process, and may have unique inputs and outputs. Using drop-down lists, users may select which output variables will serve as inputs for plugins down the line. For example, the function selected in Fig 7C takes the input variable “BW (1)”, and outputs “thin (2)” and “spineSearchZone (2)”, both of which are used as inputs in other steps down the line. A number referencing the analysis step is attached to each variable name to avoid errors where multiple plugins have outputs with the same name. To clarify the types of input and output variables associated with each plugin, as well as the general function of the plugin itself, an informational window previews all relevant information as each function is selected (Fig 7D). Once the custom segmentation process is run, all individual output variables are previewed as images in a separate window (Fig 7E). Once users are satisfied with their plugin configuration, the configuration can be saved, shared, commented on, and even rated for success at a certain task by multiple users (Fig 7F).

## Results and discussion

**Fig 8:**
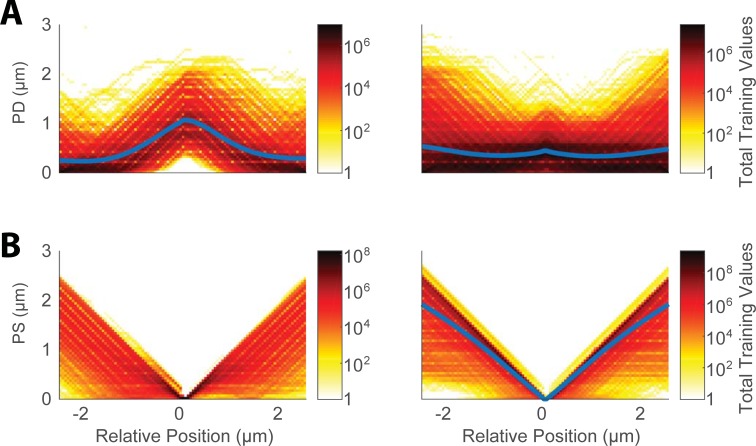
Cumulative PD and PS features. Each chart represents total points binned from all feature vectors in. (A) Left–Spine, Right–Non-Spine binned feature vectors. (B) Left–Spine, Right–Non-Spine binned feature vectors. Blue lines represent the average of all feature vectors in each group.

We used 1837 images to train, validate, and test the neural network. 3627 and 11922 feature sets were categorized as spine and non-spine, respectively. Spine PD feature arrays were often marked with a pseudo-linear increase, and then a decrease, indicating the protruding shape of the spine, while non-spine PD arrays tended to be flat, with a lower amplitude at the center (Figs 4C and 8A). PS feature arrays, on the other hand, tended to have lower amplitudes when associated with a spine, and had a more pronounced V-shape at non-spine positions (Figs 4C and 8B).

As expected, the length of the spine backbone tended to be longer in spines versus non-spines (Fig 5B). Spine IS feature arrays often had a pronounced increase followed by decrease in amplitude, indicating the bright center of the dendritic spine, while non-spine groups were characterized by a more linear drop-off in signal (Fig 5A).

**Fig 9:**
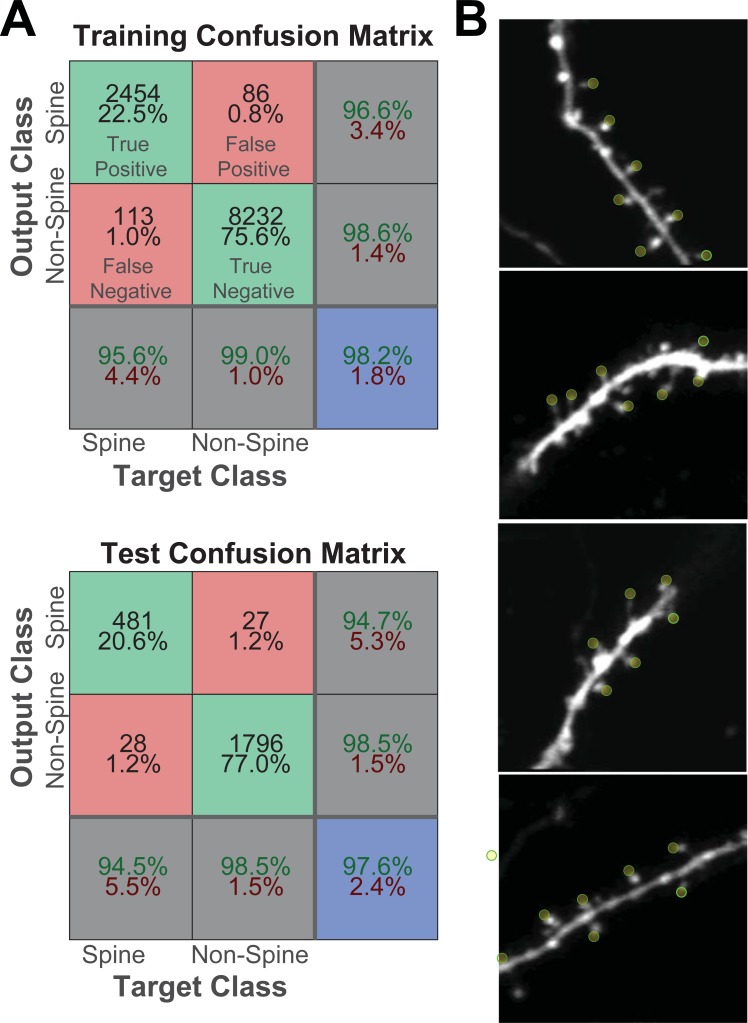
Neural Network’s ability to identify spines. (A) Training and Test confusion matrices. (B) Yellow circles indicate spines identified by neural network in naïve images.

Labeled data was split into three groups–Training (60%), Validation (20%), and Testing (20%). Classifier results for training and testing data are shown in Fig 9. Overall, classification accuracy was highly similar between training, test, and validation datasets, indicating that there was no overfitting of the model. In the testing dataset, 94.5% of actual spines were classified as spines (true positive rate), and 5.5% were classified as non-spines (false negative). 98.5% of non-spines were classified as non-spines (true negative) and 1.5% classified as spines (false positive). The positive predictive value of the algorithm (precision) was 94.7%. These results indicate that our model is highly successful in spine identification. Reshuffling of the training, validation, and testing sets did not make any significant difference to the algorithm’s success. Previous algorithms tend to report results that are either lower or as good. For example, Blumer at al. [5] achieved a true positive rate and precision of 55% and 65%, respectively, on 2P images, while Cheng et al. [7] saw results comparable to ours. Notably, many of these previous algorithms report test results on extremely small datasets, suggesting a high probability of overfitting. Algorithm functionality is also difficult to compare since some code is built specifically for images collected by other imaging tools, such as SEM, or using specific biomarkers. None of the data from our validation group was used to train the model indicates the high predictive value of our algorithms. Furthermore, spine identification in dendrites collected at magnification values different from those of the training data (Fig 9B) predicts the scalability of our algorithm to broader datasets. In keeping with best community practices, we have made our code and data open to the public in an easily accessible online format.

A major benefit of our spine classification algorithm is a lack of parameters which users are required to tune. The only input required by the algorithm is the relative scale of the image in pixels/μm, which can often be extracted automatically from images saved with modern imaging software. Furthermore, we expect our algorithm to become more powerful and accurate for dendritic spine identification as more training data becomes available. Therefore, we see our algorithm as a particularly user-friendly option for those looking to automate fluorescent imaging and/or targeting of dendritic spines. In particular, we expect that the combination of this technique with our previously developed spine imaging automation software [14] will lead to significant increases in the throughput of spine imaging and stimulation.

It is important to note that while our algorithm lacks tunable parameters, it differs significantly from the current state-of-the-art, end-to-end convolutional networks. Our model includes automated denoising and thresholding steps which are explicitly programmed as opposed to being learned. A common pitfall of including explicit processing steps in the pipeline is poor parameter tuning, which leads to imprecise feature selection, but we felt that our algorithm performs adequately enough that these problems were generally avoided. The benefit of including these initial steps over an end-to-end approach is an overall reduction in feature amount, which leads to smaller processing power for network training. Therefore, while deep, convolutional networks are an invaluable tool in computer vision, we feel that a shallow network with explicit feature selection is simple, effective, and practical, making it a useful tool for the identification of fluorescent subcellular features such as dendritic spines.

While many techniques have been developed to identify dendritic spines [5–11, 22], many of these techniques were specifically designed for post-hoc analysis, relying on additional human input to correct mistakes. While our algorithm does not claim to have 100% accuracy, its goal is to identify a large majority of clearly demarcated spines, specific to a single focus plane, within a sample for automated imaging and photostimulation. For such an automated system to work, spine labeling must require no human input, and have a minimal number of false positives, which lead to throwaway data. Our algorithm accomplishes precisely this feat, relying only on machine learning and previously labeled training data. Furthermore, to minimize the amount of human training time necessary to train the neural network algorithm, we’ve taken steps to simplify our data as much as possible, reducing images to a set of feature vectors which convey important information about spine shape. Since our training data and code is open-sourced and shared online, we expect other labs to build upon and improve our algorithm by adding their own training data, therefore increasing the potential accuracy of spine identification. Furthermore, our software can add additional training features, allowing for even further improvements of detection accuracy.

## Conclusion

Overall, we believe that our neural network model for automated spine identification in fluorescent neurons is highly accurate, scalable, and is built to easily be upgraded with the addition of training data and programmatic improvements. Due to its open-source availability, simplicity, and lack of tunable features, we expect this software to be used both in post-hoc spine analysis, as well as for automated spine tracking during imaging experiments.
